# A retrospective study: platinum-based induction chemotherapy combined with gemcitabine or paclitaxel for stage IIB-IIIA central non-small-cell lung cancer

**DOI:** 10.1186/1477-7819-11-76

**Published:** 2013-03-21

**Authors:** Chao Lv, Yuanyuan Ma, Nan Wu, Shi Yan, Qingfeng Zheng, Yu Sun, Shaolei Li, Jian Fang, Yue Yang

**Affiliations:** 1 Department of Thoracic Surgery II, Key Laboratory of Carcinogenesis and Translational Research (Ministry of Education), Peking University Cancer Hospital & Institute, Peking, China; 2 Department of Pathology, Key Laboratory of Carcinogenesis and Translational Research (Ministry of Education), Peking University Cancer Hospital & Institute, Peking, China; 3 Department of Thoracic Medical Oncology II, Key Laboratory of Carcinogenesis and Translational Research(Ministry of Education), Peking University Cancer Hospital & Institute, Peking, China

**Keywords:** Non-small-cell lung cancer, Induction chemotherapy, Central disease, Resection, Squamous

## Abstract

**Background:**

Several encouraging phase III clinical trials have evaluated platinum-based induction chemotherapy against stage IIB-IIIA non-small-cell lung cancer (NSCLC). Chemotherapy efficacy was assessed using common regimens in this retrospective analysis.

**Methods:**

From 2007 to 2011, the clinical records of stage IIB-IIIA NSCLC patients undergoing surgery after neoadjuvant chemotherapy were reviewed. Gathered data were tested for significance and variables impacting survival were assessed by univariate and Cox regression analyses.

**Results:**

Overall, 84% of patients were male and 93% had central disease. Platinum-based chemotherapy protocols with gemcitabine or paclitaxel gave an overall response rate of 55% (45/82) and 6.1% pathological complete response (5/82). Clinical response was unassociated with regimen or histology, while more pneumonectomies were performed in the stable compared to partial response disease group (*P* =0.040). Postoperative mortality was 1.2% (1/82), and complications, unassociated with regimen or histology, were atelectasis (26.8%) and supraventricular arrhythmias (13.4%). Right-sided procedures appeared to increase the incidence of bronchopleural fistula (*P* =0.073). The median disease-free survival time was 18 months and median overall survival time was not reached. Disease-free survival rates at one, two, and three years were 54%, 47%, and 33%, while the overall survival rate was 73%, 69%, and 59%, respectively. Disease-free survival predictors were radiographic response and mediastinal lymphadenopathy before chemotherapy (*P* =0.012 and 0.002, respectively).

**Conclusions:**

Two cycles of platinum-based chemotherapy with gemcitabine or paclitaxel is efficacious for patients with stage IIB-IIIA central disease. Patients achieving clinical response had improved disease-free survival times, while those with mediastinal lymphadenopathy had a higher postoperative recurrence risk.

## Background

Lung cancer in stage IIB-IIIA generally has an unfavorable prognosis, with poor five-year survival rates ranging from 19 to 25% for those using clinical stage prediction and 24 to 36% for those using pathological stage prediction, respectively [1]. Complete surgical resection has a low survival and high recurrence rate, although effective systemic therapies, including perioperative chemotherapy, radiation and combined modality therapies, have shown potential benefits in numerous clinical trials.

Following surgery, neoadjuvant chemotherapy has the potential advantage of reducing tumor volume and eradicating micrometastatic disease, thereby improving the outcome. However, the Medical Research Council (MRC) LU22 trial in 2007, which evaluated the role of induction chemotherapy prior to surgery, failed to show evidence of a difference in overall survival (OS) between neoadjuvant chemotherapy and surgery alone. In this randomized multicenter trial, 519 patients with stages IA-IIIB lung cancer, of whom 61% had stage IA or IB disease, received either surgery alone or a combination of surgery with one of six chemotherapy regimens [2]. Three years later, the Southwest Oncology Group (SWOG) 9900 trial, aimed at evaluating the efficacy of preoperative paclitaxel and carboplatin, achieved a trend toward improved OS (Hazard Ratio = 0.79) and disease-free survival (DFS) (HR = 0.80) in the induction chemotherapy arm [3]. The (Neo)Adjuvant Taxol/Carboplatin Hope (NATCH) trial included 624 patients with stage IA, IB, II, or IIIA(T3N1) NSCLC. This study finally demonstrated that no statistically significant differences in DFS were found with the addition of either preoperative or adjuvant chemotherapy to surgery [4]. However, a meta-analysis of 13 randomized control trials published in the same year concluded that neoadjuvant chemotherapy would improve the OS of operable NSCLC patients, including patients with stage III disease [5]. The recently published Chemotherapy in Early Stages Trial (ChEST), yielded a significant improvement in both DFS (HR = 0.51) and OS (HR = 0.42) in patients with clinical stage IIB-IIIA NSCLC using preoperative gemcitabine plus cisplatin [6].

Based on the above findings, we retrospectively reviewed all cases of clinical stage IIB-IIIA NSCLC whose treatment included a platinum-based induction therapy prior to surgery at our center, from the past five years, in order to comprehensively analyze the efficacy, potential complications and impact on survival of this treatment modality.

## Methods

### Patients and methods

We retrospectively reviewed the clinical records of patients who underwent surgery for NSCLC following neoadjuvant chemotherapy at Peking University Cancer Hospital from January 2007 to December 2011. All the patients had been diagnosed with NSCLC by means of biopsy, and a pretreatment evaluation was performed which included a chest computed tomography (CT) scan, a magnetic resonance imaging (MRI) scan of the brain and a bone scan in addition to an ultrasound examination of the abdomen and supraclavicular lymph nodes. Positron emission tomography - computed tomography (PET/CT) scans were not routinely used. By searching our database, patients were selected for this study based on the following features: (1) presence of central disease with T2bN1, T3 or T4 N0 to N1, or locally advanced disease with T1 to T3 N2; (2) had undergone at least two cycles of platinum-based chemotherapy preoperatively; (3) had no advanced disease such as N3 or M1; and (4) had not received radiotherapy as induction therapy. Clinical variables were recorded in detail including age, sex, histological type, clinical TNM stage, disease location, chemotherapy regimen, clinical response, type of resection, postoperative complications, final pathological TNM stage, disease recurrence and survival. Specifically, long-term survival and follow-up data were obtained from either electronic medical records or the medical statistics office. If survival or death could not be confirmed, a telephone interview was carried out in order to obtain the information.

### Treatment

All patients received platinum-based chemotherapy in one department of the hospital. Treatment consisted of gemcitabine (1,250 mg/m^2^ on days one and eight) followed by cisplatin (75 mg/m^2^on day one) or carboplatin at a dose to achieve an area under the concentration-time curve (AUC) of 5 mg/mL on day one, with treatments being repeated every 21 days. Another commonly used regimen was paclitaxel (175 mg/m^2^ on day one) combined with cisplatin or carboplatin at the same dose as described above. A further neoadjuvant chemotherapy regimen was pemetrexed (500 mg/m^2^ on day one) followed by cisplatin.

Response to the induction treatment was evaluated using CT scans at least two weeks after chemotherapy. The efficacy was classified as complete response (CR), partial response (PR), stable disease (SD) or progressive disease (PD) according to the World Health Organization (WHO) criteria. The presence of lymphadenopathy in the mediastinum (cN2) is defined as lymph nodes greater than 2.0 cm on the long axis view or 1.0 cm on the short axis view. A thoracotomy was performed after four to six weeks by the same surgical team, on the thoracic unit, after the completion of induction chemotherapy. Systematic mediastinal lymph node dissection, rather than lymph node sampling, consisted of levels 2, 3, 4, 7, 8, 9, 10 and 11 on the right as well as 4, 5, 6, 7, 8, 9, 10, 11 on the left. We also resected lobar nodes (level 12), segmental nodes (level 13) and subsegmental nodes (level 14) for accurate staging. Complete resection (R0) was defined as free resection margins with systematic nodal dissection showing the highest mediastinal node being negative, with no extracapsular nodal extension of the tumor. The final pathological examination and analysis was carried out in the same hospital department, and pathological complete response (pCR) was defined as no viable tumor cells in the specimen, as determined by light microscopy. Any complications after the operation were carefully recorded.

### Statistical analysis

Statistical analysis was performed using patched SPSS 17.0 for Windows software (SPSS Inc., Chicago, IL, USA). Data were tested for significance with the χ^2^ or Fisher’s exact test for discrete variables, and with the *t*-test for continuous variables. OS and DFS were estimated using the Kaplan-Meier method. The impact on survival of variables was assessed using univariate analysis in order to select related clinical variables and the log-rank test and Cox regression analysis to identify the effect of the covariates. Results of analyses were considered significant at a level of *P*< 0.05.

## Results

Between 2007 and 2011, 98 patients underwent surgery following neoadjuvant chemotherapy, of which 82 cases were considered for inclusion in this retrospective analysis. Of the 16 patients excluded, six were classified as N3 or M1 at diagnosis, six received induction regimens other than cisplatin or carboplatin, and two patients received just one treatment cycle preoperatively; a further two patients were excluded due to having received induction radiotherapy. The clinical characteristics of the selected 82 patients prior to treatment are given in Table 1. In general, this cohort of patients was male with central squamous carcinoma commonly diagnosed by bronchoscopy. Most patients received two cycles of gemcitabine (55%) or paclitaxel (35%), in combination with cisplatin (82%) or carboplatin (18%). Resections consisted of 16 pneumonectomies (20%), 52 lobectomies (63%), eight bilobectomies (10%) and two wedge resections (2%). A thoracotomy and exploration at the time of operation was performed on a further four patients (5%) due to unresectability or pleural dissemination. Of the patients who received resections, 20 underwent sleeve lobectomy or bronchoplasty, two underwent carinal resection with right pneumonectomy, eight underwent angioplasty of either the pulmonary artery or superior vena cava, and another seven patients underwent broncho-angioplastic (sleeve) lobectomies. R0 resection was documented in 76 patients (93%); the remaining patients had R1 or R2 resection. The treatment regimens are shown in Table 2.

**Table 1:** Patient characteristics before treatment

**Characteristic**	**Value**
Age, years	
median	57
range	38 to74
Gender	
male	69(84%)
female	13(16%)
Location of the tumor	
central	76(93%)
peripheral	6(7%)
Biopsy method	
bronchoscopy	55(67%)
CT-guided needle biopsy	20(24%)
mediastinoscopy	3(4%)
EBUS-TBNA	4(5%)
Cell type by biopsy	
squamous	47(57%)
adenocarcinoma	26(32%)
non-small-cell	9(11%)
Clinical stage	
IIB (T3N0M0/T2bN1M0)	32(39%)
IIIA (T1-3N2M0/T3N1M0/T4N0-1M0)	50(61%)

**Table 2:** Treatment characteristics

**Characteristic**	**Value**
Neoadjuvant chemotherapy	
platinum	
cisplatin	67(82%)
carboplatin	15(18%)
second agent	
gemcitabine	45(55%)
paclitaxel	29(35%)
pemetrexed	8(10%)
Treatment cycles	
2	77(94%)
3	3(4%)
>3	2(2%)
Type of resection	
pneumonectomy	16(20%)
lobectomy	52(63%)
bilobectomy	8(10%)
wedge resections	2(2%)
exploration	4(5%)
Completeness of resection	
R0	76(93%)
R1	1(1%)
R2	5(6%)
Cell type after operation	
squamous	51(62%)
adenocarcinoma	29(35%)
large cell	2(3%)

No patients died intraoperatively but one patient died within 30 days of surgery due to respiratory failure following a bronchovascular sleeve resection: the overall postoperative mortality was 1.2%. Regarding postoperative morbidity, the most common complications were atelectasis (26.8%) and supraventricular arrhythmias (13.4%). Bronchopleural fistulas were recorded in three patients: one of whom underwent right pneumonectomy, one underwenta right middle and lower lobectomy, and one had a right lower lobectomy. Other complications included one case each of chylothorax, empyema and pneumonia. There were no hemothoraces requiring reexploration or cases of pulmonary embolism after surgery, and the total postoperative morbidity was 45.1%. An analysis of complications associated with the different neoadjuvant chemotherapy regimens, type of resection, and laterality was performed. No apparent differences in complications were noted between patients who received gemcitabine or paclitaxel combined with platinum, nor was the type of surgery a significant factor. However, a right-sided procedure was associated with an increased trend towards bronchopleural fistula, although the difference was not statistically significant (*P*= 0.073) (data shown in Table 3).

**Table 3:** Complications associated with possible risk factors

**Variables**	**Complications**		
	**Atelectasis**	**Arrhythmias**	**Bronchopleural fistula**
Laterality			
right side	9	5	3
Left side	13	6	0
*P*-value	0.859	0.831	0.073
Induction regimen			
gemcitabine	11	5	1
paclitaxel	10	4	2
*P*-value	0.431	0.731	0.557
Type of resection			
pneumonectomy	NE^a^	3	1
lobectomy	NE	6	2
*P*-value	NE	0.387	0.513

NE^a^: not evaluable as ipsilateral atelectasis could not occur.

**Figure 1:**
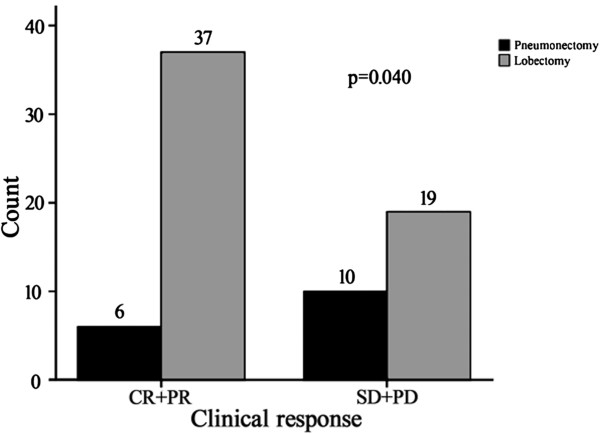
**Among 72 patients with central disease from NSCLC**, **43 patients achieved radiographic response.** Of these patients, only six underwent pneumonectomy, which was significantly fewer than those with stable disease(6/43 versus 10/29, *P* =0.040).

The radiographic response of the primary tumor to induction chemotherapy was evaluated. CR was observed in 11% of patients (9/82), while PR was noted in 44% of patients (36/82), resulting in an overall response rate of 55%. 37 patients with SD and no PD were assessed. Pathological response was evaluated with five pCR being confirmed, including four CR and one PR by clinical evaluation. The pCR rate was 6.1%. The response to treatment with the two major chemotherapy schedules (gemcitabine and paclitaxel) was not significantly different (*P* = 0.309). Subset analyses are summarized in Table 4 and showed no significant difference in response rate using gemcitabine, paclitaxel and pemetrexed for the major histological types (squamous and adenocarcinoma), (*P*= 0.572, 0.517, respectively). Seventy-two patients were identified with central disease and underwent resection surgery (10 cases were excluded either due to having undergone exploration only or to the presence of peripheral disease). Among these patients, 43 had a radiographic response (PR/CR), while 29 patients had stable disease. There was no significant difference in T size before chemotherapy between the two groups (*P*= 0.739), although more pneumonectomies were performed in the patients with stable disease (*P* = 0.04) (Figure 1).

**Table 4:** Clinical response rate and pCR rate with different regimens

**Variables**	**Radiographic response**				
	**CR/PR**	**SD/PD**	**RR**^**a**^	***P*-value**	**pCR**
All patients(n=82^b^)	45	37	55%		5(6%)
gemcitabine	24	21	53%		2(4%)
paclitaxel	18	11	62%	0.309	3(10%)
squamous (n=51)	29	22	57%		4(8%)
gemcitabine	18	14	56%		
paclitaxel	11	8	58%	0.572	
adenocarcinoma (n=29)	15	14	52%		1(3%)
gemcitabine	6	6	50%		
paclitaxel	6	3	67%		
pemetrexed	3	5	38%	0.517	

^a^RR: response rate.

^b^Note: Eight of the 82 patients who received pemetrexed were excluded in the comparison between gemcitabine and paclitaxel because pemetrexed was used only in patients with adenocarcinoma.

**Figure 2:**
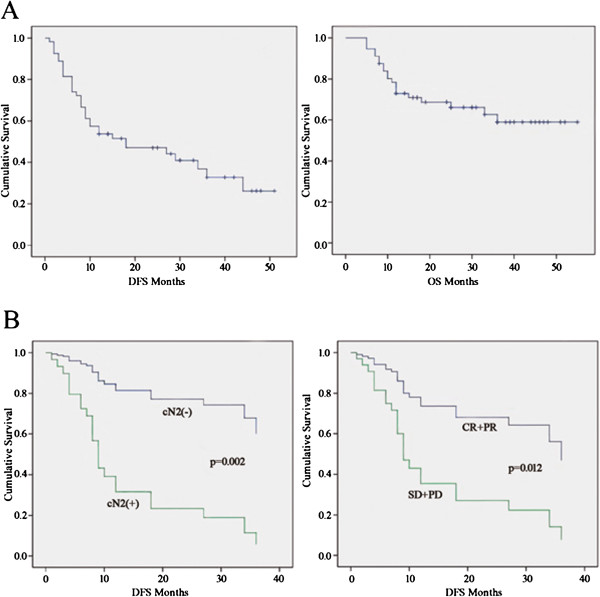
**(A) Kaplan-Meier DFS, median DFS time was 18 months (95% cumulative survival 0.6 to 35.4 months), the DFS rate at one, two, and three years was 54%, 47%, and 33%, respectively (left panel).** Kaplan-Meier OS, median OS time was not reached, the OS rate at one, two, and three years was 73%, 69%, and 59%, respectively (right panel). (**B**) DFS differences in cN2 (left panel) and clinical response (right panel) after adjustment for other factors.

Among the 82 patients, 54 had a follow-up time of more than 18 months and these were evaluated. The median follow-up time was 38.6 months, with a median DFS time of 18 months (95% cumulative survival, 0.6 to 35.4 months)and median OS time was not reached during the study's follow-up period (Figure 2A). The DFS rate at one, two, and three years was 54%, 47%, and 33%, while the OS rate was 73%, 69%, and 59%, respectively. In a univariate analysis, the effects of several factors on DFS were evaluated, including age, sex, histological type, neoadjuvant chemotherapy regimen, radiographic response and radiographic T size before chemotherapy, lymphadenopathy in the mediastinum before chemotherapy (cN2), radiographic T size after chemotherapy, type of resection, pathological mediastinal lymph node metastasis (pN2) and pathological lymph node metastasis (pN1/N2). Three univariate predictors of DFS were identified: histological type, neoadjuvant chemotherapy regimen and mediastinal lymphadenopathy (cN2). Male patients, a clinical response to therapy, T size less than 30 mm after chemotherapy, and pathological N0, all showed improved DFS times. Univariate predictors with *P*-values of at least 0.25 were entered into a multivariate model. Ultimately, we identified radiographic response (*P* = 0.012), along with cN2 (*P*= 0.002) as predictors of DFS. The HRs, respective 95% confidence intervals (CI) and *P*-values are summarized in Table 5. As shown in Figure 2B, the survival curves demonstrate significant differences between cN2+ and cN2- (left panel) and between CR+PR and SD+PD (right panel).

**Table 5:** **Univariate and multivariate predictors of disease**-**free survival (DFS)**

**Variable**	**Subset**	**HR**	**95% CI**	***P*-value**
Univariate analysis				
age	>60			
	≤60	0.845	0.425 to1.680	0.632
sex	male			
	female	2.133	0.954 to4.769	0.065
histological type	squamous			
	nonsquamous	2.191	1.088 to4.409	0.028
induction regimen	gemcitabine			
	paclitaxel	0.466	0.227 to0.957	0.038
T size before chemotherapy	>5cm			
	≤5cm	0.645	0.289 to1.440	0.285
cN2	(+)			
	(-)	0.186	0.073 to0.474	0.0004
clinical response	CR/PR			
	SD/PD	0.654	0.329 to1.301	0.226
type of resection	lobectomy			
	pneumonectomy	1.403	0.576 to3.419	0.456
T size after chemotherapy	>3cm			
	≤3cm	0.561	0.245 to 1.287	0.173
pN status	N2			
	N0/N1	1.249	0.833 to1.873	0.283
pN status	N0			
	N1/N2	1.647	0.804 to3.373	0.172
Multivariate analysis				
cN2	(+)			
	(-)	0.179	0.060 to0.531	0.002
clinical response	CR/PR			
	SD/PD	0.295	0.114 to0.766	0.012

## Discussion

Central lung cancer with or without mediastinal lymphadenopathy is a problem that thoracic surgeons continue to face. Direct resection of the tumor is challenging, incurring a high risk of massive hemorrhage and the discovery of intraoperative unresectability. In our retrospective review, 93% of patients had central disease (76/82) of whom 48% (39/82) had, at diagnosis, involvement of central great vessels such as the pulmonary artery, pulmonary vein or superior vena cava. 35% (29/82) of patients had atelectasis or the possibility of requiring pneumonectomy if resected directly. In a survival analysis, it has been proven that complete resections have a favorable impact on survival compared to incomplete resections [7], even in the case of mediastinal invasion T4 disease [8]. Surgery also offers improved local control rates and OS when compared to radiotherapy following induction therapy [9,10], indicating that R0 resection remains the cornerstone of treatment for NSCLC. The complete resection rate in our study was 93%, which is similar to that found in previous trials (77.4 to 93%) [3,7,11,12]. Although this is a retrospective study and the results would be diminished if the overall intent-to-treat (ITT) population were considered, this high R0 resection rate is still promising for cases of locally advanced central disease after chemotherapy. Clearly, patients with the above features present more intraoperative technical challenges to the thoracic surgeon and require more careful postoperative care from surgeons who have considerable experience in handling this patient population [13]. In our study, the 1.2% overall postoperative mortality is encouraging and lower than that previously reported (3 to 5%) [3,6,7]. Postoperative complications were acceptable and there were no differences observed between chemotherapy regimens and the types of resection. All three bronchopleural fistula cases occurred in right-sided procedures. This is consistent with previous results where neoadjuvant chemotherapy when followed by a right-sided procedure, especially a pneumonectomy, is an important causal factor of bronchopleural fistula [14]. We conclude that the local intrinsic anatomy may be responsible for the fistula if the procedure included a right lower lobectomy, while induction therapy is another factor that hampers bronchial stump healing.

**Figure 3:**
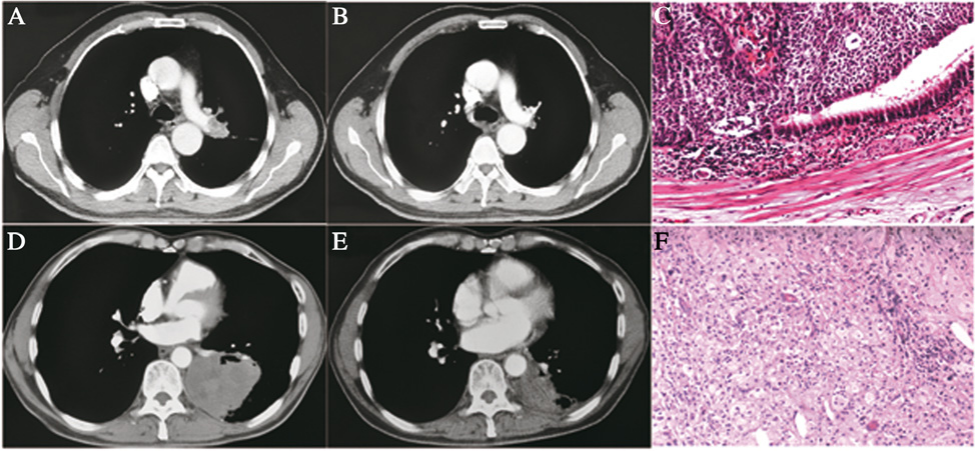
**Tumor in the left upper lobe (A) presented a CR after two cycles of induction chemotherapy with gemcitabine plus cisplatin (B), although pathological examination found residual viable tumor by light microscopy (C).** Another tumor in left lower lobe (**D**) achieved a PR after two cycles of paclitaxel plus cisplatin induction therapy (**E**), while no cancer cells were seen in the residual specimen, and all the tumor was replaced by abundant foamy cells and lymphocytic infiltration (**F**).

We observed a comparable response rate of 55% and a pCR rate of 6.1%, which are within the ranges of those reported [6,7,15-17]. The pCR rate is considered to be an important predictor of survival in patients receiving induction chemotherapy followed by surgical resection [18]. However, the reliability of radiographic CR to predict pCR is disappointing, with a high false negative rate of approximately 50% by CT and 30% by PET/CT [19]. In our study of nine cases with radiographic CR, only four (44%) were confirmed pCR with another one case confirmed from the radiographic PR cohort. This implies that surgical resection cannot be avoided following induction therapy since the rate of complete clearance of tumor using chemotherapy is low and difficult to predict preoperatively (Figure 3). Gemcitabine and paclitaxel were two major regimens used as induction therapy in our study which, in previous studies, had given good response rates for preoperative chemotherapy, ranging from 35 to 70.2% [6,17,20] and 41 to 56% [3,4,20], respectively. Our analysis showed similar results with a 53% response rate with gemcitabine and 62% with paclitaxel. In addition, we compared these regimens in different histological types, finding no significant difference in response rate. This result is consistent with that reported in The Eastern Cooperative Oncology Group (ECOG) 1594 trial and other retrospective analyses of advanced disease aimed at comparing efficacy by histological type using different regimens [21-23]. This suggests the choice of induction regimen cannot be made by considering tumor histological type alone, and further studies are required to match candidates with the appropriate regimen in order to achieve the expected response rate. Ribonucleotide reductase M1 (RRM1) and thymidylate synthase (TS) mRNA levels were once reported to be predictive of disease response and considered useful parameters for the treatment selection of gemcitabine or pemetrexed. This observation suggested promising potential for further research in this field [24].

**Figure 4:**
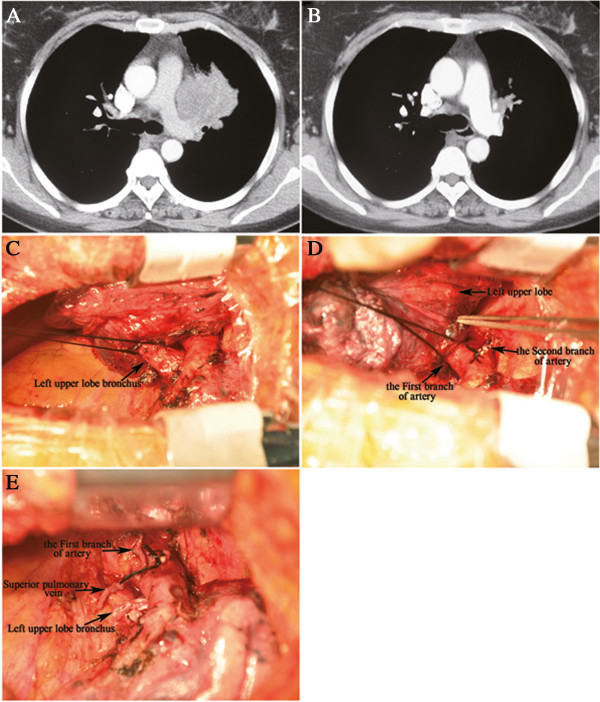
**This patient with left upper lobe lung cancer had a high risk of pneumonectomy if resected directly due to involvement of pulmonary artery (A).** However, after two cycles of induction chemotherapy, the tumor achieved significant remission (**B**). During the isolation of the upper lobe bronchus and the first branch of pulmonary artery, no significant invasion was encountered at the proximal portion (**C**, **D**). Finally, the procedure of lobectomy was performed without angioplasty or bronchoplasty (**E**).

**Figure 5:**
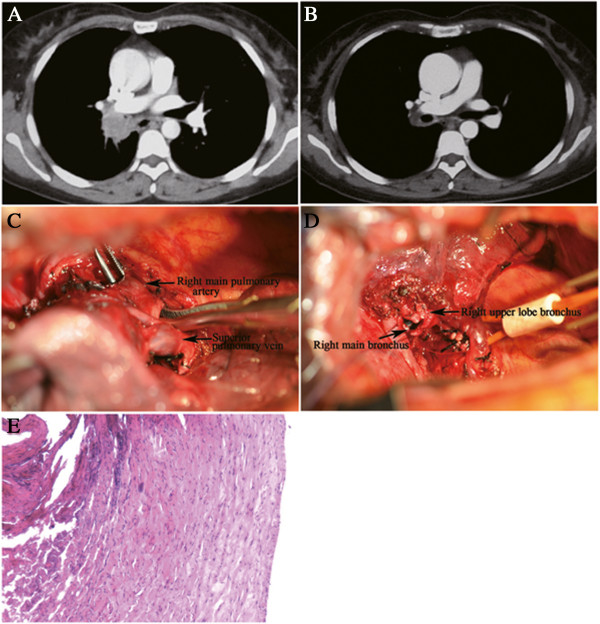
**To resect this centrally located tumor is also challenging (A), but two cycles of gemcitabine combined with cisplatin made it possible to preserve lung tissue and avoid pneumonectomy (B).** During the resection, the right main pulmonary artery was successfully isolated and controlled (**C**). Finally, the procedure of right middle and lower lobectomy with bronchoplasty was performed (**D**), and the arterial margin was found to be free of tumor (**E**).

**Figure 6:**
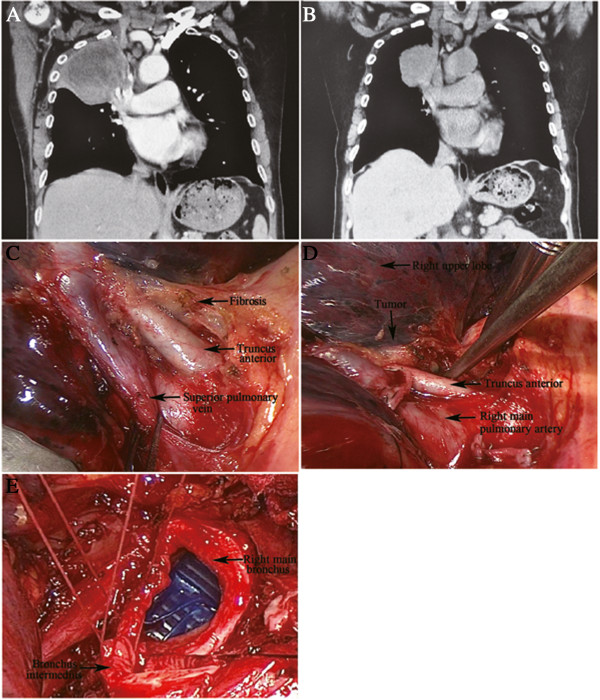
**The upper right pleural cavity was occupied by a large squamous carcinoma**, **and the right hilum had also been invaded (A).** After neoadjuvant chemotherapy, the tumor shrank significantly (**B**). Although the induction therapy created fibrosis in the hilum (**C**), the truncus anterior was exposed and isolated successfully (**D**). Finally, the patient underwent right upper lobe sleeve resection without angioplasty (**E**).

Whether and to what extent neoadjuvant chemotherapy could change the type of resection or even enable treatment of unresectable disease remains controversial and unclear in most published studies. Disease evaluation is based on radiographic images which are subjective and highly dependent on the experience and expertise of the thoracic surgeon [13], and the type of surgery cannot be determined until an intraoperative exploration is performed. MRC-LU22 once reported that a small proportion of patients (about 5%) were able to undergo a lobectomy rather than a pneumonectomy as a result of neoadjuvant chemotherapy [2]. In all cases of central disease that had been resected, we observed that the number of successful pneumonectomies was significantly higher in the SD group than in the CR or PR group (*P*= 0.040). Two cases of unresectable disease occurred in the former group, which supports the possible theory that potential pneumonectomies for unresectable disease could be avoided in favor of bronchoplasty or angioplasty procedures by means of efficacious preoperative chemotherapy. Although a clinical trial designed to confirm this function of induction therapy would not be possible, we believe that induction chemotherapy facilitates and simplifies the surgery of patients with central disease as illustrated in Figures 4, 5, and 6. Many studies have reported that pneumonectomy was one of the major factors influencing outcome in patients with locally advanced NSCLC after induction chemotherapy [14,15,25]. Statistically significant differences were observed in postoperative mortality for pneumonectomies (11.3%) compared with lobectomies (2.4%), especially for right pneumonectomies [26]. Furthermore, a low compliance rate in accepting postoperative adjuvant chemotherapy was observed in patients who underwent a pneumonectomy [27]. Therefore, in our center, given the considerable abnormalities in the respiratory and cardiovascular systems in our patients, those patients who had had a pneumonectomy did not receive adjuvant chemotherapy or radiotherapy due to the potential toxicity risk of these treatments. Despite this, neither excess morbidity nor shortened DFS were found in this cohort; findings which we attributed to good postoperative care, reasonable patient selection and appropriate induction chemotherapy.

The OS rates of 73% at one year and 59% at three years were within the range of those reported by previous studies which included all the pathological stages [3,6,12]. These represent the reality of clinical practice in our center. Because the median OS time was not reached in our study, we focused on DFS time which is closely linked to surgery and perioperative therapy. In previous retrospective studies, it has been demonstrated that age, complete resection, pneumonectomy and pathological stage all had a significant impact on survival [7,15,16,25]. In addition to these factors, mediastinal downstaging is another important determinant of survival, particularly for stage IIIA disease [16,28]. During our study, downstaging was not considered due to the unreliable evaluation of clinical nodal (cN) status by CT. We noted a trend toward difference in DFS for pathological nodal (pN) status, although this was not statistically significant (*P*= 0.172). However, we observed that patients displaying central disease with mediastinal lymphadenopathy had a high postoperative risk of recurrence unless a pN0 status was achieved with induction chemotherapy.

## Conclusion

This is a retrospective review of a group of patients characterized by having central disease from clinical stage IIB-IIIA non-small-cell lung cancer and who had received two cycles of platinum-based chemotherapy prior to surgical resection. Although the study was potentially limited due to the small number of patients, short follow-up period and unreliable N staging before chemotherapy, we achieved a comparable response rate and pCR rate with a high R0 resection and low mortality rate, demonstrating the feasibility and efficacy of this relatively simple but standard treatment model. The good clinical response might have had some influence on avoiding pneumonectomy and unresectability. Additionally, we found that radiographically stable disease, together with cN2, was considered an adverse factor for DFS.

## Consent

Written informed consent was obtained from the patient for publication of this report and any accompanying images.

## Abbreviations

AUC: Area under the concentration-time curve; CR: Complete response; CT: Computed tomography; DFS: Disease-free survival; ITT: Intent-to-treat; NSCLC: Non-small-cell lung cancer; OS: Overall survival; pCR: Pathological complete response; PD: Progressive disease; PET/CT: Positron emission tomography - computed tomography; PR: Partial response; RRM1: Ribonucleotide reductase M1; SD: Stable disease; TS: Thymidylate synthase

## Competing interests

The authors declare that they have no competing interests.

## Authors’ contribution

SY, QFZ, YS, JF and CL participated in the design of the study, performed the statistical analysis and drafted the manuscript. YYM and NW participated in study design, literature search and coordination. SY, QFZ, YS and JF participated in the analysis of experimental results. YY conceived of the study, participated in its design and coordination, and helped to draft the manuscript. All authors read and approved the final manuscript.
